# SMETANA: Accurate and Scalable Algorithm for Probabilistic Alignment of Large-Scale Biological Networks

**DOI:** 10.1371/journal.pone.0067995

**Published:** 2013-07-12

**Authors:** Sayed Mohammad Ebrahim Sahraeian, Byung-Jun Yoon

**Affiliations:** 1 Department of Plant and Microbial Biology, University of California, Berkely, California, United States of America; 2 Department of Electrical and Computer Engineering, Texas A & M University, College Station, Texas, United States of America; Semmelweis University, Hungary

## Abstract

In this paper we introduce an efficient algorithm for alignment of multiple large-scale biological networks. In this scheme, we first compute a probabilistic similarity measure between nodes that belong to different networks using a semi-Markov random walk model. The estimated probabilities are further enhanced by incorporating the local and the cross-species network similarity information through the use of two different types of probabilistic consistency transformations. The transformed alignment probabilities are used to predict the alignment of multiple networks based on a greedy approach. We demonstrate that the proposed algorithm, called SMETANA, outperforms many state-of-the-art network alignment techniques, in terms of computational efficiency, alignment accuracy, and scalability. Our experiments show that SMETANA can easily align tens of genome-scale networks with thousands of nodes on a personal computer without any difficulty. The source code of SMETANA is available upon request. The source code of SMETANA can be downloaded from http://www.ece.tamu.edu/~bjyoon/SMETANA/.

## Introduction

The complicated interactions among numerous cellular constituents – such as DNAs, RNAs, and proteins – govern numerous complex cellular functions. For instance, protein-protein interactions (PPI) conduct various transcriptional, signaling, and metabolic processes in cells [1]. Graphical representation of these complex interactions, where biomolecules are represented as nodes and their interactions as edges, can help us better understand and investigate the structure and dynamics of diverse biological mechanisms [2], [3]. Thanks to the recent technological advances in high-throughput interaction measurement techniques, along with many text-mining tools developed to search the biomedical research literature for known molecular interactions, large-scale PPI networks are currently available for a number of model organisms, and biological network databases are still undergoing rapid expansion [4]–[8]. Availability of such large-scale interaction data has expedited comprehensive studies of biological networks, and the development of accurate and efficient computational techniques for network analysis is expected to lead to the discovery of novel biological knowledge. Cross-species comparison of genome-scale PPI networks can serve as one effective way of analyzing the available biological networks [9], [10]. As demonstrated in many comparative genome studies, such a comparative approach can provide effective computational framework for identifying functional modules (e.g., signaling pathways or protein complexes) that are conserved across different networks [9].

One of the research problems that are actively studied in the field of comparative network analysis is the network alignment problem. The main goal of network alignment is to predict the best mapping between two (or more) networks, based on the similarity of the constituent molecules and their interaction patterns. By investigating the cross-species variations of biological networks, network alignment may be employed for predicting conserved functional modules [11] or inferring the function of unannotated proteins [12]. To obtain biologically meaningful alignment results, the network alignment algorithm needs to integrate the similarity between the individual nodes (i.e., biomolecules in the networks) – in terms of their composition, structure, or function – as well as the similarity between their interactions patterns (i.e., topological similarity). As shown by a reduction to the graph isomorphism problem [13]–[15], the optimal network alignment problem is NP-hard. Therefore, many comparative network alignment schemes impose additional mathematical constraints or adopt various heuristics to make the problem computationally feasible [14]–[39].

While most of these schemes were developed for pairwise network alignment, several schemes have been proposed for the more challenging problem of aligning *multiple* networks [16]–[19], [21], [22]. As the complexity of the problem grows exponentially with the number of networks to be aligned, multiple network alignment algorithms need to devise a simple and scalable alignment scheme so that they can be used for aligning more than just a few networks. NetworkBLAST-M [21], [22], which is one of the pioneering network alignment algorithms, greedily searches for highly conserved local regions in the alignment graph constructed from the potential orthologous nodes. Using a progressive scheme, Græmlin [16], [17] successively performs pairwise alignment of the closest network pairs by maximizing an objective function based on a set of learned parameters. IsoRank [19] greedily builds up the multiple network alignment according to the pairwise node similarity scores computed using spectral graph theory. IsoRankN [18] further extends the idea in IsoRank by employing a spectral clustering scheme. As the number of networks in the alignment increases, the overall computational cost tends to sharply increase and the alignment quality tends to decrease for most existing schemes, making them impractical for aligning multiple large-scale networks.

In this paper, we propose a novel method, called SMETANA (**S**emi-**M**arkov random walk scores **E**nhanced by consistency **T**ransformation for **A**ccurate **N**etwork **A**lignment), for finding the maximum expected accuracy (MEA) alignment of large-scale biological networks. In this scheme, we first compute the node correspondence scores based on a semi-Markov random walk model. These scores can be efficiently computed using a closed-form formula and they provide a probabilistic similarity measure between nodes that belong to different networks. To effectively incorporate the similarities across multiple networks, we additionally employ two different types of probabilistic consistency transformations that can enhance the initial node correspondence scores, originally obtained from pairwise network comparison. The transformed scores are subsequently used to construct the MEA global alignment in a greedy manner. To demonstrate the effectiveness of SMETANA, we extensively evaluate its performance based on real and synthetic examples. We show that SMETANA clearly outperforms state-of-the-art network alignment techniques, in terms of computational efficiency, alignment accuracy, and scalability.

## Materials and Methods

Suppose we want to align a set of 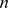 PPI networks 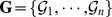. Each network 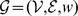 consists of a set 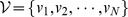 of *N* nodes that correspond to the proteins in the network; a set 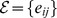 of 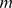 undirected edges that represent the protein interactions, where the edge 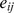 denotes the interaction between proteins 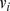 and 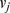; and a weight function 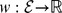, representing the strength or reliability of an interaction. Then, for any two networks 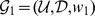 and 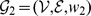, we show the node similarity score for a pair of proteins 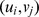, where 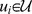 and 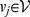, as 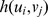. Typically, sequence similarity scores are used to measure this node similarity, although it is possible to use other measures based on structural or functional similarity between the proteins.

### Estimation of probabilistic node correspondence scores through semi-Markov random walk

An effective network alignment scheme should map protein nodes across the given PPI networks based on their overall biological similarity, measured by integrating the node similarity (e.g., sequence-based similarity) between the matching proteins as well as the similarity between their patterns of interactions with the neighboring proteins. The semi-Markov random walk (SMRW) model provides an effective means of estimating such integrated similarity scores [40], [41]. Markov random walk is a process that consists of a succession of random steps (on a graph or a path) according to the Markov assumption. Unlike an ordinary Markov random walk, in which the random walker always spends a fixed amount of time between each transition, in a semi-Markov random walk, the walker may spend a random amount of time between the moves. To estimate the node correspondence scores, we consider a simultaneous semi-Markov random walk on a pair of networks as in [41] by taking simultaneous random steps on both networks. This simultaneous random walk on two graphs 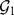 and 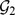 is equivalent to a random walk on their product graph 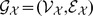 [41], [42]. Based on this model, we define the *global correspondence score* 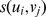 between any two nodes 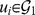 and 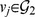, as described in the Supporting Information S1.

### Estimation of the Pairwise Node Alignment Probabilities

Here, we aim to employ the computed SMRW correspondence scores to define the likelihood of alignment between each node pair. We represent the pairwise node alignment probability between any two nodes 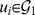 and 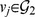 as 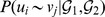. To compute such a probability, we exploit the correspondence scores obtained in the previous step, as follows:
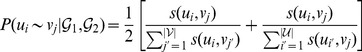
(1)


This way, we consider the relative importance of 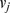 for matching with 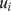 with respect to other homologues of 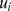 in 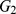, and vice versa. This consideration is an important issue, since a meaningful posterior alignment probability should balance the alignment likelihood across the network, assign relative priority to the nodes, and remain symmetric with respect to the pair networks (i.e. 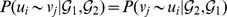). This can be written in a simple matrix form as follows:
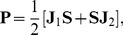
(2)where 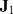 is a 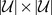-dimensional diagonal matrix such that 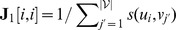, 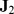 is an 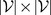-dimensional diagonal matrix such that 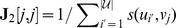, and 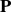 is a 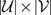-dimensional matrix such that 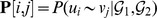.

### Enhancing Probability Estimation Through Consistency Transformations

Here, we use two types of probabilistic consistency transformations to improve the pairwise node alignment probabilities 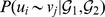 using the information from neighboring nodes in the original pair of networks as well as the alignment information from other networks in the alignment. This modification makes these probabilities suitable for constructing a consistent and accurate multiple network alignment.

#### Intra-network probabilistic consistency transformation

In the first consistency transformation, we incorporate the information from the neighboring nodes to update the original pairwise node alignment probabilities. This transformation is motivated by the observation that nodes are mostly conserved across networks as connected complexes or pathways. Thus, if most of the neighbors of 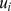 are aligned to most of the neighbors of 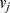 then there is a high chance that 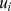 will be aligned to 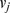. Therefore, we can utilize the neighbors' alignment probabilities to better estimate the alignment probability of a given node pair 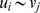. Based on this motivation, we introduce the intra-network probabilistic consistency transformation defined as follows:
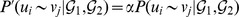


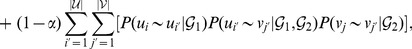
(3)where 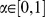 is a parameter that determines the balance between the original pairwise alignment probability and the influence from the neighbors, and 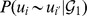 is the probability that 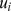 will be a neighbor of 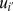. The transformation in (3) can be written in a matrix form as follows:
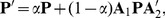
(4)where 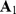 and 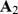 are the transition probability matrices of 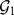 and 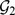, respectively, and 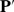 is a 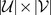-dimensional matrix such that 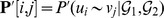. To avoid false positives, we only update 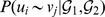 to 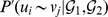 for those node pairs that satisfy 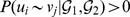 or if 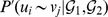 is among the top 1% highest alignment transformed probabilities in the network.

#### Cross-network probabilistic consistency transformation

In the second consistency transformation, we incorporate the information from other networks in the alignment to improve the estimation of pairwise node alignment probabilities. The proposed probabilistic consistency transformation is motivated by a similar idea that has been utilized in multiple sequence alignment, which was based on the motivation that all the pairwise alignments induced from a multiple alignment should be consistent with each other [43]. For example, given three networks 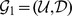, 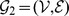, and 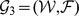, if 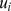 (a node in 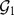) aligns with 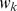 (a node in 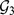) in the projected 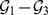 alignment, and at the same time, if 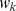 aligns with 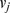 (a node in 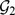) in the projected 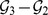 alignment, then 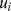 should also align with 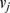 in the projected 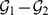 alignment. Thus, we can utilize the “intermediate” network 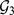 to improve the estimate of 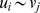 alignment probability by incorporating the consistency information in 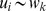 and 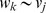 alignments. Here, we extend the idea of the improved probabilistic consistency transformation proposed in [44] for multiple sequence alignment to the multiple network alignment problem at hand. This transformation considers the relative significance of each intermediate network in improving the pairwise alignment probabilities.

Let 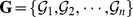 represent the set of *n* networks to be aligned. We define 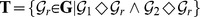 as the set of networks in **G** that are related to both 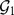 and 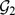, where 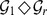 means 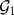 and 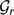 are homologous. Using only the networks in **T**, we define the following probabilistic consistency transformation:
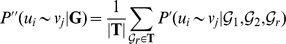


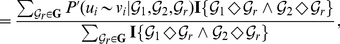
(5)where 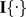 is the identity function which checks whether 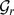 is homologue to both 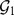 and 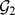, and 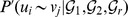 is the node alignment probability given the three networks 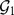, 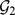, and 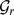. As in the multiple sequence alignment case [43], 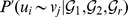 can be approximated as 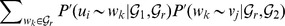.

In practice, we cannot judge with certainty whether two networks are homologous or not. Thus, taking the expectation of (5) along with the independence assumption of 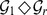 and 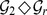, we obtain:
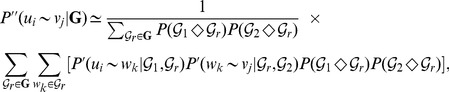
(6)where 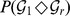 is the probability that 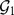 and 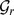 are homologous to each other. We estimate this probability as
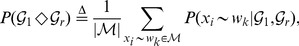
(7)where 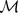 is maximum weighted matching of 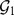 and 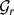. We can rewrite (6) in a matrix form as follows:
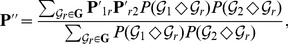
(8)where 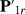 is the transformed matrix of 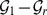 alignment computed as in (4). Similarly, 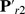 is the transformed matrix of 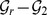 alignment.

As before, to avoid false positives, we only use this transformation to update the alignment probability of node pairs with non-zero alignment probability, or if their transformed probability is within the top 1%.

#### Alignment construction

Given a set of networks **G**, our ultimate goal is to find the multiple network alignment that maximizes the expected accuracy (i.e. the expected number of correctly aligned nodes) over all networks in **G**. Let 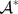 be the true (unknown) alignment. We define accuracy of the alignment 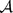 with respect to the alignment 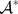 as:
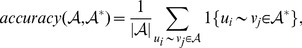
(9)which is the relative proportion of correctly matched nodes. Since the true alignment is not known, we seek to maximize the expected accuracy of the alignment as proposed in [43]:
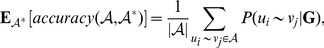
(10)where 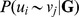 is the posterior probability of 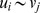 alignment. If we use the consistency transformed probabilities, defined in (6), as the alignment probability of node pairs, the MEA problem will reduce to the standard maximum-weighted n-partite matching, which is NP-hard. Thus, we find a suboptimal solution to this problem through a greedy approach. In this scheme, we start with the null alignment, and greedily construct the alignment through successive insertion of the node pair 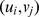 with the largest posterior pairwise node alignment probability. While growing the alignment, we consider the following two constraints to avoid false positives:

While inserting a new node pair 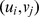 to the alignment, if only one of these nodes (e.g., 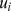) was previously included in the alignment (e.g., in the equivalence class **C**), we check if there exist other nodes in 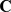 from the same network of node 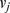. For instance, consider 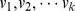 as well as 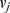 are all in network 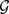, and the nodes 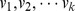 are already in the aligned group **C**. In such a case, we only consider adding 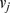 to **C** if 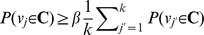, where 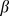 is a scaling factor and 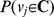 is the probability of appearance of 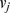 in the alignment group **C**, defined as:
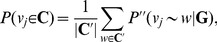
(11)where 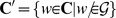 is the set of nodes in **C** from networks other than 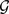.

In this way, we verify whether the coherence of 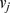 to **C** is sufficiently close to the average coherence of other nodes in **C** which are also from the same network 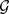.

- We also restrict the maximum number of nodes from one network in any alignment group to 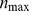.

Based on the alignment process described above, we can ultimately find the global alignment of the given set of networks. In the final alignment, each node may be mapped to several nodes that belong to other networks.

## Results

To investigate the performance of the proposed network alignment algorithm, we conducted a set of experiments based on three suites of synthetic benchmark datasets as well as a number of real PPI network examples. We compared the performance of SMETANA against four well-known multiple network alignment algorithms: IsoRankN [18], NetworkBLAST-M (NBM) [21], Græmlin 2.0 [17], MI-GRAAL [35], C-GRAAL [36], AlignNemo [37], and PINALOG [38]. In our experiments, we used the restricted-order version of NBM as the running time of the relaxed-order version increases exponentially with respect to the number of networks to be aligned. Græmlin needs to learn the parameters of its scoring function, and to this aim, we used the same training set as in [45]. We adopted the graphlet degree signature distance and the E-values (measuring the sequence similarity) as the similarity measures used in the MI-GRAAL and C-GRAAL algorithms. To test AlignNemo on synthetic data, we regarded nodes whose similarity score exceeds 100 as putative orthologues. The parameter 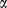, which determines the balance between sequence similarity and topological similarity, was set to 0.6 for IsoRankN as in the original paper [18]. For SMETANA we set 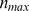 to 10, 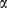 to 0.9, and 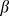 to 0.8. We use various measures to asses the specificity, sensitivity, functional consistency, coverage, and interaction conservation of network alignment algorithms, as in other studies [17], [18], [35]. We refer to the set of aligned nodes (i.e., potential orthologs) as the *equivalence class*. Each equivalence class may include an arbitrary number of nodes from each network. To compute these accuracy measures, we first remove the unannotated nodes (nodes with no functional annotations) from the alignment result and also remove equivalence classes containing only a single node. A given equivalence class is viewed as being *correct* if all the included nodes belong to the same functional group.

### Alignment Performance on NAPAbench Benchmark Dataset

We first evaluated the performance of the proposed algorithm on NAPAbench [45], an extensive alignment benchmark that consists of large-scale synthetic PPI network families. Currently, NAPAbench consists of three suites of datasets: the *pairwise alignment* dataset, the *5-way alignment* dataset, and the *8-way alignment* dataset. Each of these suites contain PPI network families generated using three different network growth models, namely, DMC [46], DMR [47], and CG [48], which enables the performance assessment of network alignment algorithms under diverse conditions. The pairwise dataset contains three network pairs, where each pair consists of a network with 3,000 nodes and another network with 4,000 nodes. The 5-way dataset consists of three network families, each with five networks with 1,000, 1,500, 2,000, 2,500, and 2,500 nodes, respectively. This dataset simulates a family of PPI networks that correspond to distantly related species. Finally, the 8-way alignment dataset also consists of three network families, each with eight networks of 1,000 nodes. The networks in each network family are obtained by evolving an ancestral network of size 400. The 8-way alignment dataset simulates network families of closely-related species. Further details about these benchmark datasets can be found in [45].

#### SPE, CN, and MNE measures

To measure the overall accuracy of the predicted alignments, we first computed the following measures for SMETANA as well as previous network alignment algorithms:

Specificity (SPE): The relative number of correctly predicted equivalence classes.

Correct Nodes (CN): The total number of nodes (i.e., proteins) that are assigned to the correct equivalence class. This measure reflects the sensitivity of the prediction [17].

Mean normalized entropy (MNE): The mean normalized entropy of the predicted equivalence classes can provide an effective measure of the consistency of the predicted network alignment. The normalized entropy of a given equivalence class **C** can be computed by:
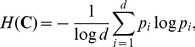
(12)where 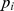 is the fraction of proteins in **C** that belongs to the 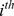 functional group, and 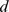 is the number of different functional groups. That is, a cluster that consists of nodes with higher functional consistency will have lower entropy.

The SPE, CN, and MNE of different network alignment algorithms on the pairwise, 5-way, and 8-way datasets are respectively summarized in Tables 1, 2, and 3 for the DMC, DMR, and CG datasets in NAPAbench. As we can see in Table 1, for the pairwise alignment, NBM has the highest specificity and the lowest entropy, while SMETANA yields significantly higher number of correctly aligned nodes (i.e., CN), implying its higher sensitivity. However, as the number of networks increases, the NAPAbench shows clear advantage in terms of all SPE, CN, and MNE (see results for 5-way and 8-way datasets). On average, SMETANA shows around 10% improvement in SPE, 30% improvement in CN, and 35% improvement in MNE over the next best algorithm (IsoRankN) on the 5-way dataset. The improvement is even higher for the 8-way alignment, SMETANA leads to 25%, 60%, and 40% improvements in terms of SPE, CN, and MNE. Since NBM algorithm only predicts equivalence classes that are conserved across all the compared species (i.e. they have at least one node from each network), we also report the accuracy of each network alignment algorithm in predicting equivalence classes that are conserved across all networks. These results are shown in the last three rows of Table 2 and Table 3. Interestingly, this comparison shows that SMETANA outperforms NBM, as well as the other algorithms, even by a larger margin. Experimental results in this section clearly demonstrate that SMETANA can effectively track the similarity between nodes across multiple networks, while previous algorithms show performance degradation as the number of networks increases.

**Table 1:** Performance of different algorithms for pairwise network alignment.

	DMC			DMR			CG		
	SPE	CN	MNE	SPE	CN	MNE	SPE	CN	MNE
SMETANA	92.58	**5191**	6.93	91.48	**4933**	7.39	94.80	**4889**	4.81
IsoRankN	82.69	3836	14.13	83.55	3915	13.40	83.16	3868	13.34
NBM	**96.55**	3185	**4.98**	**96.75**	2853	**4.02**	**96.23**	4523	**4.03**
Græmlin 2.0	77.37	2137	15.70	81.03	2322	13.33	90.72	2549	7.96
MI-GRAAL	66.13	3612	35.27	69.97	3852	31.59	79.48	4385	22.76
C-GRAAL	32.12	1779	66.52	43.80	2430	55.74	63.34	3523	37.56
AlignNemo	77.37	2137	15.70	81.03	2322	13.33	90.72	2549	7.96
PINALOG	70.64	3707	30.79	71.57	3735	29.83	71.66	3935	29.84

Performance comparison based on the pairwise alignment of two networks of size 3,000 and 4,000. The performance of each method is assessed using the following metrics: specificity (SP), number of correct nodes (CN), and mean normalized entropy (MNE). In each column, best performance is shown in bold.

**Table 2:** Performance of different algorithms for 5-way network alignment.

	DMC			DMR			CG		
	SPE	CN	MNE	SPE	CN	MNE	SPE	CN	MNE
SMETANA	**91.21**	**7299**	6.94	**91.55**	**7203**	7.13	**93.60**	**7359**	5.51
IsoRankN	80.91	5538	10.27	79.58	5496	11.14	82.68	5689	9.72
NBM	85.17	1038	5.40	79.32	1182	6.81	84.62	1995	4.64
Græmlin 2.0	51.07	3028	16.32	50.88	3100	16.94	62.89	4451	13.19
SMETANA (only 5-species)	89.07	4067	**4.64**	88.93	3712	**4.43**	92.17	3782	**2.66**
IsoRankN (only 5-species)	69.67	1859	9.67	68.07	1610	10.26	73.83	2223	7.99
Græmlin 2.0 (only 5-species)	35.90	1575	19.50	36.60	1581	20.29	54.44	2394	14.17

Performance comparison based on the 5-way alignment of five networks of size 1500, 2000, 2500, 3000 and 3000. The last three rows are obtained by considering only equivalence classes that contain at least one node from every species. The performance of each method is assessed using the following metrics: specificity (SP), number of correct nodes (CN), and mean normalized entropy (MNE). In each metrics, best performance is shown in bold.

**Table 3:** Performance of different algorithms for 8-way network alignment.

	DMC			DMR			CG		
	SPE	CN	MNE	SPE	CN	MNE	SPE	CN	MNE
SMETANA	87.04	**6349**	7.15	86.07	6207	7.53	89.69	6485	5.88
IsoRankN	64.50	4069	13.62	62.52	3938	14.58	61.18	3890	14.58
NBM	80.38	643	5.51	72.95	881	7.78	87.63	1264	3.24
Græmlin 2.0	58.67	2315	16.51	51.34	1939	19.38	49.29	2729	17.24
SMETANA (only 8-species)	**92.12**	3686	**3.81**	**90.77**	3358	**3.59**	**95.95**	3784	**1.60**
IsoRankN (only 8-species)	56.74	1987	10.06	54.36	1797	10.81	54.30	2172	10.33
Græmlin (only 8-species)	13.08	345	29.83	9.87	291	31.63	25.66	802	20.78

Performance comparison based on the 8-way alignment of eight networks of equal size 1,000. The last three rows are obtained by considering only equivalence classes that contain at least one node from every species. The performance of each method is assessed using the following metrics: specificity(SP), number of correct nodes (CN), and mean normalized entropy (MNE). In each column, best performance is shown in bold.

#### Coverage

**Figure 1:**
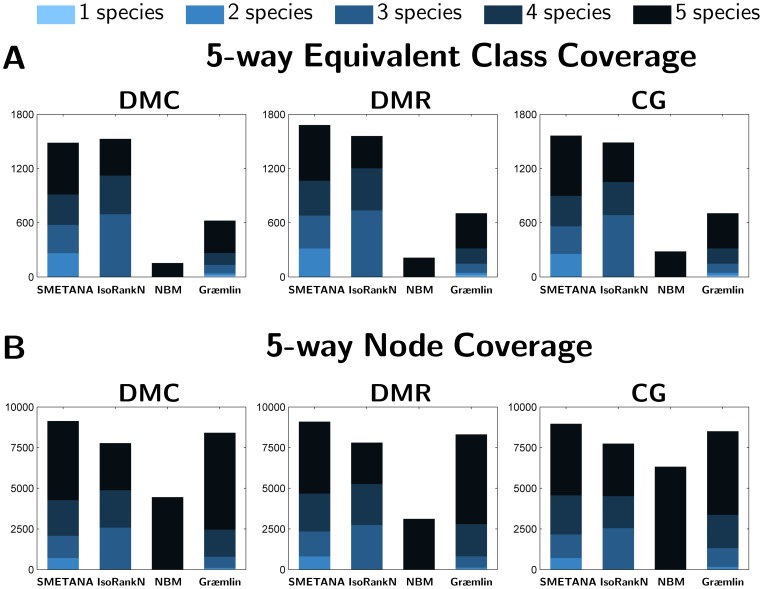
Performance of various network alignment algorithms. (A) Equivalence class coverage: Number of equivalence classes in the 5-way alignment experiment that contain nodes from 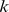 species (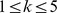). (B) Node Coverage: Number of nodes (i.e. proteins) that belong to equivalence classes that contain nodes from 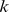 species in the 5-way alignment.

**Figure 2:**
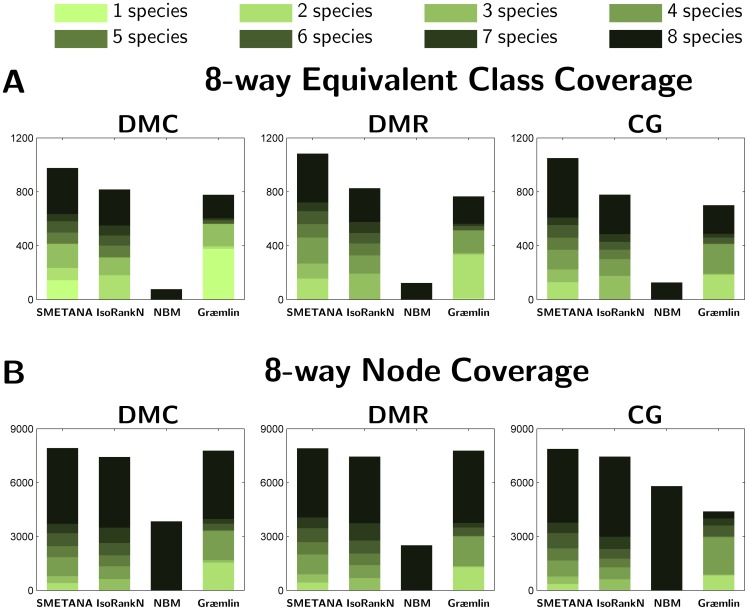
Performance of various network alignment algorithms. (A) Equivalence class coverage: Number of equivalence classes in the 8-way alignment experiment that contain nodes from 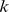 species (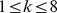). (B) Node Coverage: Number of nodes (i.e. proteins) that belong to equivalence classes that contain nodes from 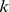 species in the 8-way alignment.

Next, we investigate the coverage of the predicted equivalence classes in the 5-way and the 8-way datasets. We report the coverage in terms of two measures. The first measure is the number of predicted classes that consist of nodes from 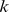 different networks, where 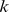 ranges between 1 and 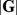 (i.e., the total number of networks in the dataset). As another measure of coverage, we report the total number of nodes (i.e proteins) in the predicted classes. As before, we split the number of predicted nodes based on the number of different species in the equivalence class they belong to. Results for 5-way alignment are shown in Figure 1. As we can see, SMETANA and IsoRankN predict a larger number of equivalence classes, where SMETANA predicts about 50% more classes that contain nodes from all 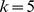 networks. In terms of the number of predicted nodes, we can also observe that the SMETANA results in better coverage compared to other algorithms and that most of the predicted nodes belong to equivalence classes that span 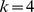 or 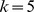 species. The above results show that SMETANA yields multiple network alignments with better coverage without sacrificing the alignment accuracy (e.g., see Table 2). Besides, considering that the 5-way alignment dataset consists of networks with varying size, we expect to have equivalence classes with 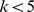 species. This implies that the restriction in the NBM algorithm to report only equivalence classes with 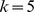 species may be too stringent when comparing the networks of remotely related species and it may result in lower alignment accuracy. In fact, this can be seen in Table 2, where the NBM yields lower SPE and CN, and higher MNE scores. Similar trends can be observed from the 8-way alignment results, as shown in Figure 2. SMETANA also attains better coverage on this dataset compared to other algorithms and most of its predictions spans 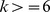 species.

#### Conserved interactions

To verify whether the predicted network alignments effectively capture the topological conservation across networks, we investigate the number of conserved interactions in the alignment results obtained using different alignment schemes. We report two metrics for this purpose. The first metric, CI (conserved interactions), reports the total number of perfectly conserved edges between all equivalence classes in the alignment. The second metric, COI (conserved orthologous interactions), reports the total number of conserved edges between “correct” equivalence classes that consist of orthologous nodes.

**Figure 3:**
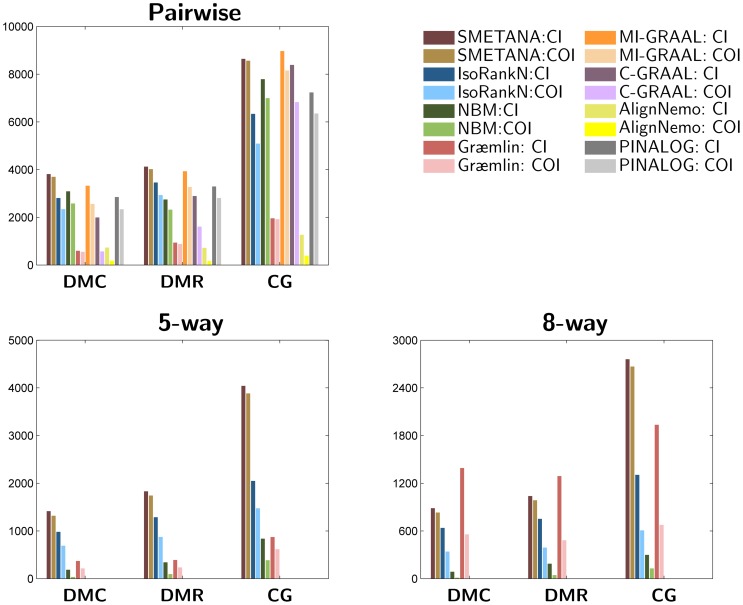
Number of conserved interactions (CI) and conserved orthologous interactions (COI) for different alignments. CI reports the total number of conserved edges between any of the equivalence classes in the alignment. COI reports the total number of conserved edges between “correct” equivalence classes that consist of orthologous nodes.

Results for the pairwise, 5-way, and 8-way alignment datasets are shown in Figure 3. For pairwise alignments, we can observe that SMETANA and MI-GRAAL lead to the largest number of conserved interactions (i.e., high CI) among the compared algorithms. However, it should be noted that more than 97% of the conserved edges predicted by SMETANA are between orthologous equivalence classes (i.e., the average ratio of 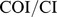 is 97%), while this ratio is around 83% for MI-GRAAL. On the 5-way and 8-way datasets, SMETANA yields network alignment results with significantly higher CI and COI compared to the other algorithms. We can also observe that around 95% of the conserved edges connect orthologous equivalence classes. In contrast, the average 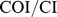 ratio is around 60% for IsoRankN and around 30% for NBM. These results suggest that the network alignments predicted by other alignment schemes may often contain spurious interactions that do not actually correspond to real conserved interactions between orthologous nodes. On the other hand, SMETANA can successfully unveil conserved interactions between orthologous proteins across multiple networks.

### Performance Dependence on Sequence Similarity

Here, we study the effect of sequence similarity on the performance of the various network alignment algorithms. To this aim, we vary the separation between the similarity score distributions of orthologous and non-orthologous nodes by a variable 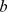 as defined in [45]. A larger 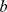 separates the two distributions further, thereby making it easier to align the networks (and to predict potential orthologs across networks) based on sequence similarity alone, without necessarily looking into their topological similarity.

**Figure 4:**
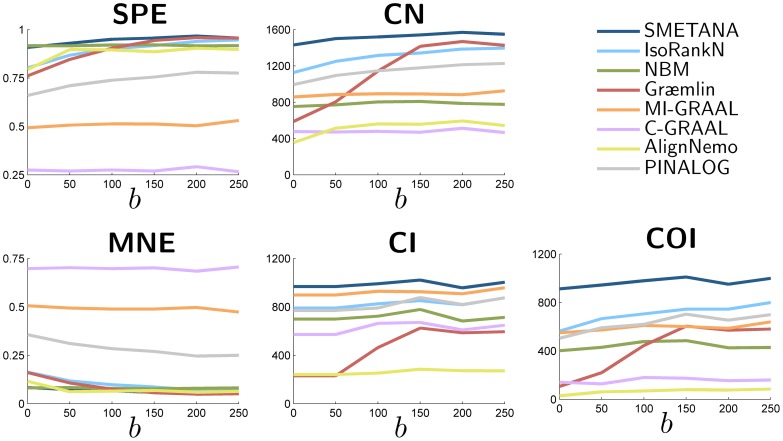
Effects of node similarity on the performance of different network alignment algorithms. The alignment performance has been estimated at several different levels of separation between the similarity score distribution for orthologous node pairs and that for non-orthologs pairs. Increasing the bias 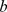 increases the separation between the two score distributions, which increases the discriminative power of the node similarity score for predicting potential orthologs. Measures reported: specificity (SPE), number of correct nodes (CN) (which reflects the sensitivity), mean normalized entropy (MNE), number of conserved interactions (CI), number of conserved orthologous interactions (COI).

For this experiment, we generated two networks, each with 1,000 nodes, from an ancestral network with 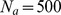 nodes. Figure 4 shows how the performance metrics change with respect to 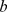. As we can see, for SMETANA, IsoRankN Græmlin, AlignNemo, and PINALOG, the overall alignment accuracy (reflected in the five performance metrics: SPE, CN, MNE, CI, COI) tends to improve as the separation between the two similarity score distributions increases. In contrast, NBM, MI-GRAAL, and C-GRAAL show more or less constant performance regardless of the separation, implying that these algorithms are less reliant on node similarity scores. Figure 4 clearly shows that SMETANA consistently outperforms other alignment algorithms in all cases and that its performance does not depend too much on sequence similarity.

### Computational Complexity

**Figure 5:**
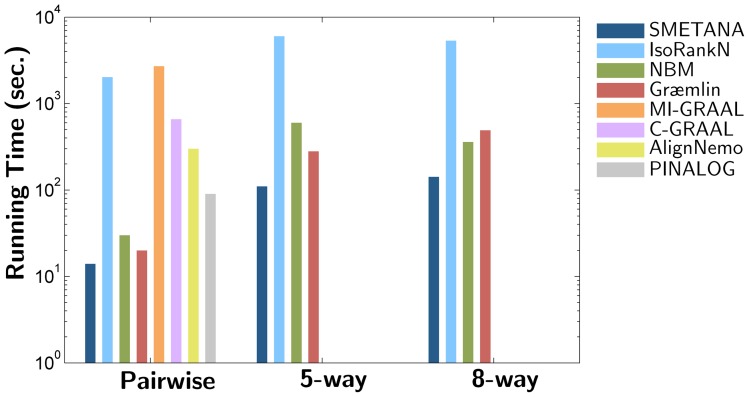
Total CPU time for aligning the networks. The total CPU time for the pairwise, 5-way, and 8-way alignments. CPU time has been averaged over DMC, DMR, and CG datasets (measured in seconds).

The proposed network alignment scheme is highly efficient, and the computational complexity of SMETANA is only polynomial in terms of the number of networks and the size of the networks. Suppose we have 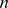 networks, where the maximum network size is 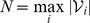, the maximum number of interactions in a network is 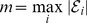, and the maximum number of non-zero elements in a pairwise similarity score matrix **H** is 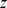. Then the overall complexity of the algorithm will be 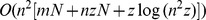. In practice, this can be approximated as 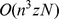. Figure 5 compares the computational complexity of different algorithms, based on the total CPU time that is needed to align the networks in the pairwise, 5-way, and 8-way alignment datasets. All experiments have been performed on a desktop computer with a 2.2 GHz Intel Core2Duo CPU and 4GB memory. It should be noted that Græmlin requires a training stage to estimate the parameters used by the algorithm, which took more than a day in our experiments. We can observe in Figure 5 that SMETANA is the fastest among the compared algorithms. In fact, SMETANA can provide alignment results in just a few minutes even for 5 or 8 large networks.

### Performance Analysis on Real Networks

Next, we conducted further experiments to verify the performance of SMETANA on real PPI networks. For these experiments, we took the PPI network of *S. cerevisiae* and generated a set of PPI network by re-sampling the original network independently. More specifically, we first randomly picked a seed node among the high-degree nodes (potential hubs) in the *S. cerevisiae* network. We then iteratively grew the network by randomly inserting 20% of the neighbors of the current network. We stopped growing the network when the total number of nodes in the network exceeded 600. The final networks typically contained around 1,000 nodes. We then used SMETANA, IsoRankN, and NBM to align 2
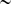
20 re-sampled networks and compared their performance. In this way, we can assess the scalability of the respective alignment algorithms and see how they perform as the number of networks grows.

**Figure 6:**
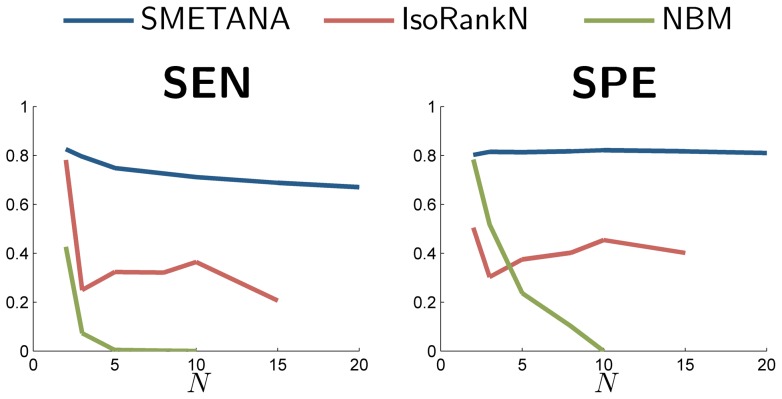
Performance on real networks. The trend of change in sensitivity (SEN) and specificity (SP) as the number of networks in the alignment increases for different multiple network alignment algorithms.

To assess the alignment accuracy, we used the KEGG orthology (KO) annotation of the proteins. A node without any KO annotation was considered to be in a correct equivalence class only if all the other aligned nodes (in the given class) correspond to the same parent node in the original PPI network of *S. cerevisiae*. Figure 6 illustrates the trends of sensitivity (the relative number of nodes that are assigned to the correct equivalence class) and specificity (the relative number of correctly predicted equivalence classes) as the number of networks in the alignment increases. As we can see, SMETANA maintains good performance, even up to 20 networks, significantly outperforming other methods. In fact, our results show that the accuracy of the other alignment schemes quickly degrades with increasing number of networks. Figure S1 shows the overall computational time that is needed to align the networks, as the number of networks increases. We can observe that NBM and SMETANA have considerably lower complexity compared to IsoRankN.

### Alignment Results Based on Real PPI Networks

**Figure 7:**
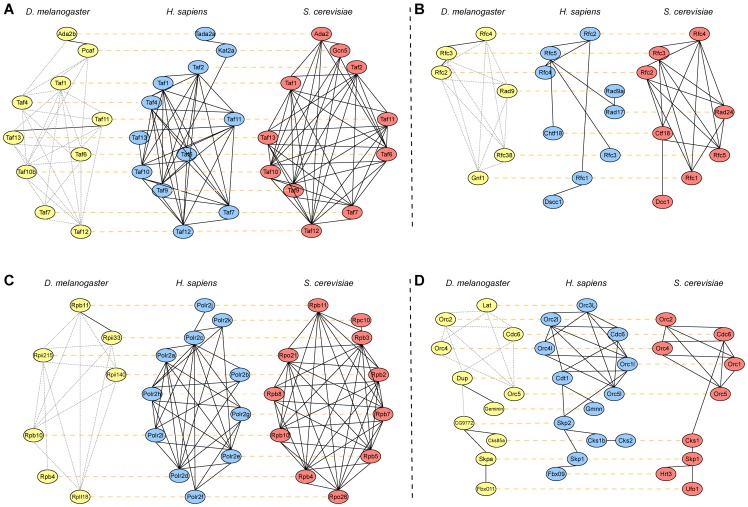
Conserved subnetwork regions in the 3-way alignment of *D. melanogaster*, *H. sapiens*, and *S. cerevisiae* using the proposed method. (A) Transcription factor. (B) Replication factor C. (C) RNA polymerase. (D) DNA replication. (Aligned nodes are placed in the same row of alignment and connected with yellow dashed lines. In each network, the interaction in the IsoBase dataset is shown in solid lines. For *D. melanogaster* some edges which are missed in IsoBase but are present in STRING protein interaction database [52] is shown in dotted gray lines).

In this section, we present some example alignment results obtained from aligning real PPI networks using SMETANA. In this experiment, we aligned the PPI networks of *D. melanogaster*, *H. sapiens*, and *S. cerevisiae*, which are the three largest PPI networks that are currently available. We obtained the PPI data from IsoBase [12], a recently published database of protein interaction networks that has been constructed by integrating the data in three different public databases: DIP [49], BioGRID [50], and HPRD [51]. Figures 7A-D show four conserved subnetworks that correspond to transcription factor, replication factor C, RNA polymerase, and DNA replication complexes, respectively. In this figure, the aligned nodes (i.e., nodes that belong to the same equivalence class) are placed in the same row and are connected with yellow dashed lines. In each network, the interactions in the IsoBase dataset are shown in solid lines. For *D. melanogaster*, some edges that are missing in IsoBase but are present in the STRING protein interaction database [52] are shown in gray dotted lines. In all of these examples, the aligned proteins predicted by SMETANA belong to the same KEGG orthology (KO) group, reflecting the high functional coherence of the predicted equivalence classes. We can also observe that SMETANA can effectively recover the conserved interactions and handle inserted/deleted nodes and interactions without difficulty.

## Discussion

In this paper, we proposed a novel network alignment algorithm, called SMETANA, that can efficiently align multiple large-scale PPI networks. The algorithm estimates the pairwise node alignment probabilities using a semi-Markov random walk (SMRW), and the estimated probabilities are updated using probabilistic consistency transformations.The transformations proposed in this paper utilize local and global similarities within and across networks, which are ultimately helpful for predicting a more consistent alignment of multiple networks. The updated node alignment probabilities are employed in a greedy alignment construction scheme, which aims to maximize the expected accuracy of the final network alignment. Extensive evaluations based on real and synthetic PPI networks clearly demonstrate that the proposed algorithm can serve as an effective tool for accurately aligning multiple networks. Especially, the proposed algorithm truly stands out when aligning a large number of networks. In fact, our simulation results show that SMETANA delivers consistently high performance as the number of networks increases. These results reflect the effectiveness of the proposed intra-network and cross-network probabilistic consistency transformations, which further enhance the pairwise node alignment probabilities that are initially estimated by the SMRW model by incorporating additional information from other networks. SMETANA is also highly efficient and scalable and it can easily align tens of networks with thousands of nodes within a few minutes on a personal computer.

## Supporting Information

Supporting Information S1 **File contains supporting method on semi-Markov random walk scores computation and Figure S1.**
(PDF)
