# Supplementary material for: SMETANA: Accurate and Scalable Algorithm for Probabilistic Alignment of Large-Scale Biological Networks
Source: PLoS One. 2013 Jul 12;8(7):e67995. doi: 10.1371/journal.pone.0067995 (PMC3710069; doi:10.1371/journal.pone.0067995)
Supplement: Supporting Information S1 — File contains supporting method on semi-Markov random walk scores computation and Figure S1. (PDF) [file pone.0067995.s001.pdf]

Supplementary Material for "SMETANA: Accurate and Scalable Algorithm for Probabilistic Alignment of Large-Scale Biological Networks"

Sayed Mohammad Ebrahim Sahraeian and Byung-Jun Yoon

Department of Electrical and Computer Engineering, Texas A&M University, College Station, TX 77843, USA

S1 Semi-Markov Random Walk scores

To measure the *global correspondence score* between any two nodes $u_i \in \mathcal{G}_1$ and $v_j \in \mathcal{G}_2$, we compute the the long-run proportion of time that the random walker stays at the node pair $x = (u_i, v_j)$ in $\mathcal{G}_\mathcal{X}$. We model the semi-Markov random walk on $\mathcal{G}_\mathcal{X}$ such that $\mu(x)$, the expected amount of time that the random walker spends at a node pair $x = (u_i, v_j)$, is proportional to the node similarity $h(u_i, v_j)$. As a result, both higher interaction similarity as well as higher node similarity between nodes would lead to higher global similarity between them. Thus, as discussed shown in [1, 2], this global correspondence score can be computed as follows:

$$\begin{aligned} s(u_i, v_j) &= \frac{\pi_\mathcal{X}(x)\mu(x)}{\sum_{x' \in \mathcal{V}_\mathcal{X}} \pi_\mathcal{X}(x')\mu(x')} \\ &= \frac{\pi_1(u_i)\pi_2(v_j)h(u_i, v_j)}{\sum_{i'=1}^{|\mathcal{U}|} \sum_{j'=1}^{|\mathcal{V}|} \pi_1(u_{i'})\pi_2(v_{j'})h(u_{i'}, v_{j'})}, \end{aligned} \quad (1)$$

where $\pi_\mathcal{X}$ is the steady state distribution of the Markov random walk on $\mathcal{G}_\mathcal{X}$, which using the decoupling property of the product graph [3], can be computed as $\pi_\mathcal{X} = \pi_1 \otimes \pi_2$, where π_1 and π_2 are the steady state distributions of the random walks on $\mathcal{G}_1$ and $\mathcal{G}_2$, respectively. We can compute these distributions by finding the eigenvectors (with unit eigenvalue) of the transition matrices of each network. We can conveniently rewrite (1) as:

$$\mathbf{S} = \frac{\mathbf{Q} \circ \mathbf{H}}{\text{trace}(\mathbf{Q}\mathbf{H}^T)}, \quad (2)$$

where $\mathbf{S}$, $\mathbf{H}$, and $\mathbf{Q}$ are $|\mathcal{U}| \times |\mathcal{V}|$-dimensional matrices such that $\mathbf{S}[i,j] = s(u_i, v_j)$, $\mathbf{H}[i,j] = h(u_i, v_j)$, and $\mathbf{Q}[i,j] = \pi_1(u_i)\pi_2(v_j)$, and $\circ$ denotes the Hadamard (or element-wise) product. We compute such correspondence score matrix for all pairs of the given networks.

An important advantage of the SMRW model is its high scalability in terms of network size. A similar random-walk-with-restart approach was originally proposed in [4] to compute functional similarity scores between nodes. As discussed in [2], a practical limitation of the scheme used in [4] is its high computational complexity of $O(m_1 \cdot m_2)$ for two networks respectively with m_1 and m_2 edges, while the SMRW scheme reduces the computational cost to $O(m_1 + m_2 + z)$ through decoupling the networks, where z is the number of non-zero elements in $\mathbf{H}$. The matrix $\mathbf{H}$ is typically sparse, rendering the complexity of the SMRW scheme significantly smaller than that in [4], especially for large networks.

References

[1] Sahraeian S, Yoon BJ (2011) A novel low-complexity hmm similarity measure. Signal Processing Letters, IEEE 18: 87 -90.

[2] Sahraeian SM, Yoon BJ (2012) RESQUE: Network reduction using semi-Markov random walk scores for efficient querying of biological networks. Bioinformatics 28: 2129–2136.

[3] Vishwanathan S, Schraudolph NN, Kondor R, Borgwardt KM (2010) Graph Kernels. Journal of Machine Learning Research 11: 1201–1242.

[4] Singh R, Xu J, Berger B (2008) Global alignment of multiple protein interaction networks with application to functional orthology detection. Proc Natl Acad Sci USA 105: 12763–12768.

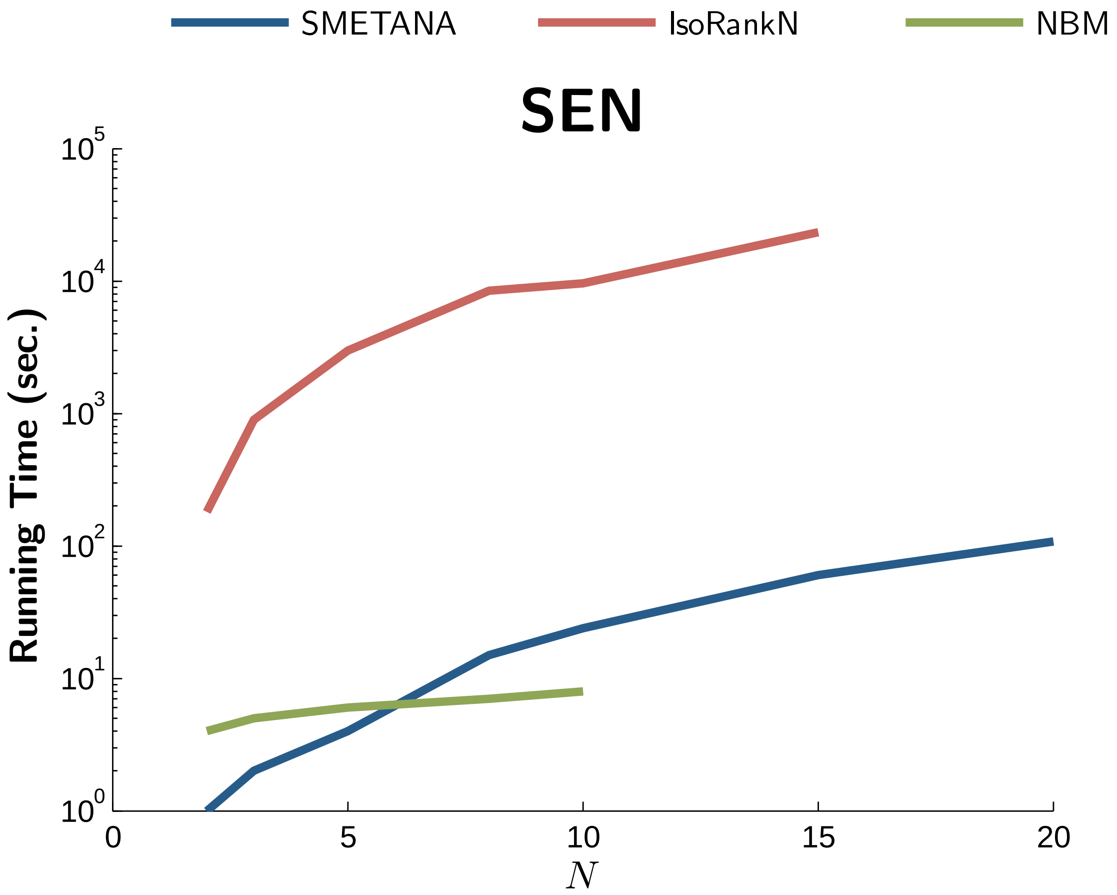

Figure S1: **Total CPU time for aligning real networks.** The trend of change in computation time as the number of networks in the alignment increases.
